# Characterization of PDGF-Induced Subcellular Calcium Regulation through Calcium Channels in Airway Smooth Muscle Cells by FRET Biosensors

**DOI:** 10.3390/bios14040179

**Published:** 2024-04-07

**Authors:** Mingxing Ouyang, Binqian Zhou, Chunmei Li, Linhong Deng

**Affiliations:** 1 Institute of Biomedical Engineering and Health Sciences, School of Medical and Health Engineering, Changzhou University, Changzhou 213164, China; 2 School of Pharmacy, Changzhou University, Changzhou 213164, China

**Keywords:** calcium signal, calcium channels, FRET biosensor, subcellular calcium regulation, platelet-derived growth factor (PDGF), airway smooth muscle cells

## Abstract

The homeostasis of cellular calcium is fundamental for many physiological processes, while the calcium levels remain inhomogeneous within cells. During the onset of asthma, epithelial and inflammatory cells secrete platelet-derived growth factor (PDGF), inducing the proliferation and migration of airway smooth muscle (ASM) to the epidermal layer, narrowing the airway. The regulation of ASM cells by PDGF is closely related to the conduction of calcium signals. In this work, we generated subcellular-targeted FRET biosensors to investigate calcium regulation in the different compartments of ASM cells. A PDGF-induced cytoplasmic calcium [Ca^2+^]_C_ increase was attributed to both extracellular calcium influx and endoplasmic reticulum (ER) calcium [Ca^2+^]_ER_ release, which was partially regulated by the PLC-IP_3_R pathway. Interestingly, the removal of the extracellular calcium influx led to inhibited ER calcium release, likely through inhibitory effects on the calcium-dependent activation of the ER ryanodine receptor. The inhibition of the L-type calcium channel on the plasma membrane or the SERCA pump on the ER resulted in both reduced [Ca^2+^]_C_ and [Ca^2+^]_ER_ from PDGF stimulation, while IP_3_R channel inhibition led to reduced [Ca^2+^]_C_ only. The inhibited SERCA pump caused an immediate [Ca^2+^]_C_ increase and [Ca^2+^]_ER_ decrease, indicating active calcium exchange between the cytosol and ER storage in resting cells. PDGF-induced calcium at the outer mitochondrial membrane sub-region showed a similar regulatory response to cytosolic calcium, not influenced by the inhibition of the mitochondrial calcium uniporter channel. Therefore, our work identifies calcium flow pathways among the extracellular medium, cell cytosol, and ER via regulatory calcium channels. Specifically, extracellular calcium flow has an essential function in fully activating ER calcium release.

## 1. Introduction

Calcium ions (Ca^2+^), as a ubiquitous messenger in cells, are one of the most important types of biochemical signals involved in many cellular physiological processes. Changes in Ca^2+^ concentrations often lead to changes in the levels of related downstream kinases, thereby regulating physiological processes such as cell differentiation [1,2], apoptosis [3], gene expression [4], etc. The calcium signal transduction in cells is precise and diverse, and similar trends may lead to different outcomes. The interaction between organelles and the action of calcium channels jointly maintains the dynamic balance of the intracellular calcium levels. Intracellular Ca^2+^ homeostasis is an important foundation for the maintenance of the normal physiological activity of cells. Abnormal calcium signaling can lead to the occurrence of disease. Asthma is a chronic airway disease, often accompanied by shortness of breath and airway remodeling during onset [5,6]. At the same time, airway epithelial cells and inflammatory cells secrete PDGF to promote the contraction and proliferation of airway smooth muscle (ASM) cells [7,8]. The regulation of ASM cells largely relies on the conduction of calcium signals [9].

In fact, the distribution of Ca^2+^ within cells is not uniform, and there is a certain concentration gradient. Among them, the endoplasmic reticulum (ER) is the largest intracellular organelle related to Ca^2+^ storage [10]. Under normal physiological conditions, the level of Ca^2+^ in the ER can reach over a thousand times the Ca^2+^ concentration in the cytoplasm [11]. Excessive or insufficient calcium levels in the ER can promote an unfolded protein reaction response and cause cell apoptosis [12]. Mitochondria also have the function of storing and releasing calcium and play an important role in the conduction of calcium signals [13]. The enrichment of calcium in the mitochondrial matrix can promote mitochondrial ATP production, but excessive calcium concentrations can also induce cell apoptosis [14]. In the cytoplasm, the increase in calcium levels is mainly due to the action of the calcium channels on the plasma membrane and the release of calcium stored in organelles. The uptake of extracellular calcium by cells mainly depends on several calcium channels to control the calcium influx on the plasma membrane, such as L-type calcium channels [15] and store-operated Ca^2+^(SOC) channels [16].

The regulation of calcium signaling by cells receiving extracellular cytokine stimulation is inseparable from the G-protein-coupled receptors (GPCRs) on the plasma membrane. GPCRs can induce the stimulation of cytokines and convert them to achieve the activation of intracellular phospholipase C (PLC) [17]. Phosphorylated PLC can further promote the production of inositol-1,4,5 tris-phosphonate (IP_3_) [18]. The IP_3_ receptor (IP_3_R) is predominantly located on the ER, and induction by IP_3_ can promote the release of calcium from the ER, leading to the upregulation of the cytoplasmic calcium levels [19,20]. In addition, there is another calcium release channel on the ER, the ryanodine receptor [21], and the ER organelles maintain high calcium levels through the SERCA pump uptake of calcium from the cytoplasm [22]. The uptake of calcium by mitochondria depends on the related calcium transporters, such as mitochondrial calcium uniporter (MCU) [23]. In addition, studies have shown that mitochondrial-associated membranes (MAMs), the contact part between the outer and ER membranes of mitochondria, are involved in calcium signaling between the ER and mitochondria [24,25].

Changes in intracellular calcium signals represent a rapidly changing process, sometimes recovering from calcium oscillations to a resting state in only a few tens of seconds. Traditional chemical fluorescent dyes for calcium verification often present inaccuracies and significant errors and cannot reflect changes in subcellular calcium levels in a timely manner. Fluorescence resonance energy transfer (FRET) is an emerging technique used to sensitively detect the spatial change between a designed fluorescent donor and acceptor, and biosensors achieve rapid results and present the ability to observe biochemical activity at the cellular or subcellular levels [26,27,28]. FRET-based calcium biosensors enable the real-time visualization of changes in intracellular and subcellular calcium levels. Calmodulin (CaM) is the core component in the construction of calcium biosensors [29]. The biosensor applies CaM to bind four free calcium ions, whose activation leads to the binding of the M13 peptide for a conformational change within the biosensor, resulting in an increase in FRET efficiency [30,31]. By adding localization peptides [32], FRET biosensors can achieve calcium detection at subcellular levels. Previously, a calcium indicator based on a single fluorescent protein with circular permutation (cpGFP) has also been developed to monitor subcellular calcium levels with high sensitivity [33].

**Figure 1:**
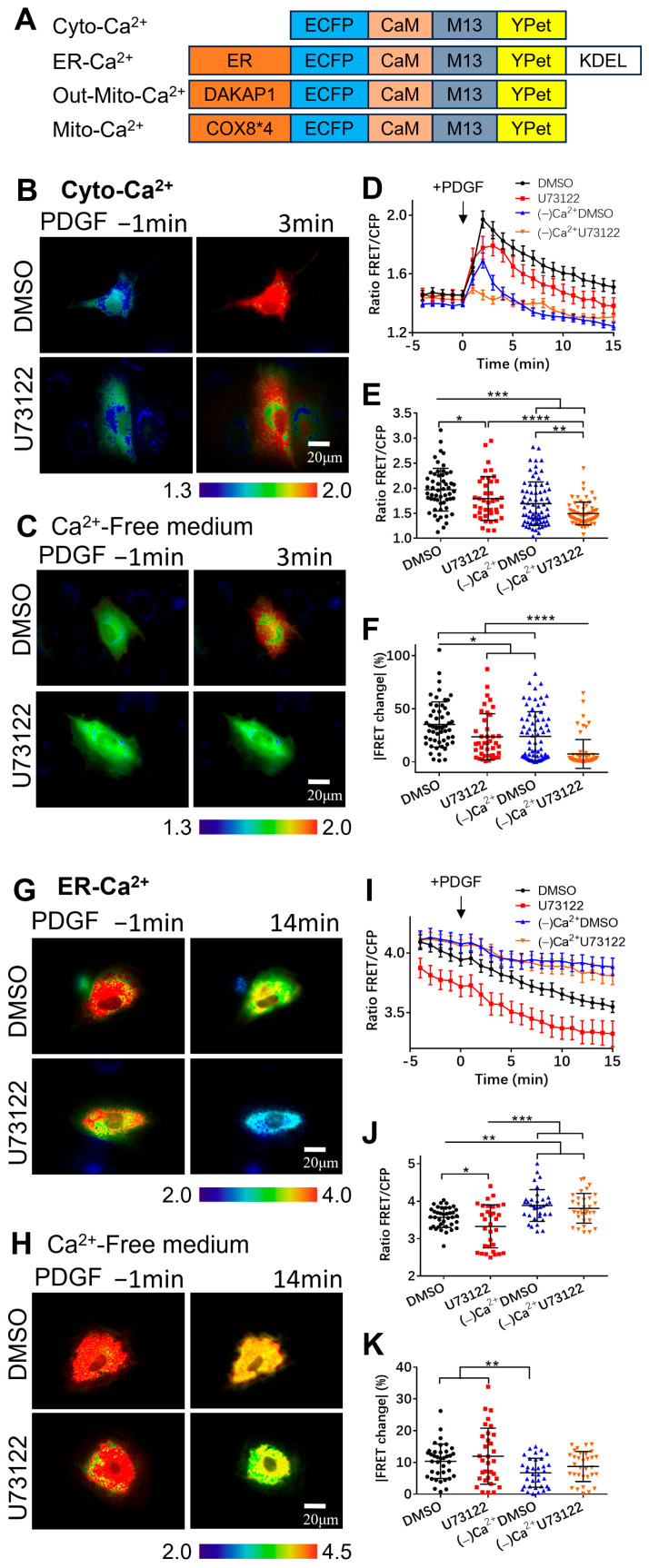
PDGF-induced calcium changes in cell cytoplasm and ER storage. The calcium concentrations were measured by calcium FRET biosensors. (**A**) Depictions of the four types of calcium FRET biosensors, as described in the Methods. (**B**,**C**) Ratiometric FRET images of the Cyto-Ca^2+^ biosensor induced with PDGF (50 ng/mL) in ASM cells pretreated with DMSO (as control) or U73122 (10 μM) in a normal culture medium (**B**) or calcium-free culture medium (**C**). (**D**) Quantified time-course curves of the cytoplasmic calcium FRET ratio (FRET/ECFP) in the ASM cells under the (**B**,**C**) conditions in a normal or Ca^2+^-free medium. (**E**,**F**) Statistical comparisons of the peak values of the FRET/ECFP ratio (**E**) and the FRET change rates (**F**) from the quantified curves in (**D**). Sample sizes of the Cyto-Ca^2+^ FRET measurements for DMSO, U73122, (−)Ca^2+^/DMSO, and (−)Ca^2+^/U73122 are 55, 40, 72, 66, respectively. (**G**,**H**) Ratiometric FRET images of the ER-Ca^2+^ biosensor induced with PDGF in ASM cells pretreated with DMSO or U73122 in a normal medium (**G**) or Ca^2+^-free medium (**H**). (**I**–**K**) Quantified time-course curves of the ER calcium FRET ratio (**I**) and statistical comparisons of the peak values of the FRET/ECFP ratio (**J**) and FRET change rates (**K**) under the various conditions of (**G**,**H**). Sample sizes of the ER-Ca^2+^ FRET measurements for DMSO, U73122, (−)Ca^2+^/DMSO, and (−)Ca^2+^/U73122 are 58, 47, 45, 47, respectively. *, **, ***, and **** indicate *p* values < 0.05, 0.01, 0.001, and 0.0001 to denote significant differences.

In this study, we used the FRET technique for the real-time monitoring of the calcium levels in the cytoplasm, ER, mitochondria outer membrane (Out-Mito), and mitochondrial matrix (mito-matrix) with four types of FRET biosensors. They were cytoplasmic calcium (Cyto-Ca^2+^), ER calcium (ER-Ca^2+^), mitochondrial outer membrane calcium (Out-Mito-Ca^2+^), and mito-matrix calcium (Mito-Ca^2+^) biosensors. These FRET calcium biosensors were proteins consisting of four core parts, namely ECFP, CaM, the M13 peptide, and YPet [34], while specific signal peptides can help to target the biosensor proteins towards subcellular locations (diagram shown in Figure 1A). In this work, by combining the FRET biosensors with several common calcium channels’ inhibitors, the PDGF-induced flow mechanism of calcium signaling in the different compartments of ASM cells was explored. Our work shows that PDGF stimulation promotes calcium influx through the plasma membrane and calcium release from the ER, leading to an increase in the cytosolic calcium level and the mitochondrial uptake of calcium. Moreover, the extracellular calcium influx through the plasma membrane affects ER calcium release, which is not entirely dependent on the PLC-IP_3_R pathway.

## 2. Materials and Methods

### 2.1. Cell Culture

The airway smooth muscle (ASM) cells used in our experiments were purchased from Beina Biotech Co. (Beijing, China), which were derived from the ASM of female Sprague Dawley rats aged 6–8 weeks. Primary rat ASM cells were cultured in a low-sugar medium (DMEM, Sigma-Aldrich, St. Louis, MO, USA) containing 10% fetal bovine serum (FBS, Thermo Fisher Scientific, Waltham, MA, USA) and maintained in a humid incubator at 37 °C with 5% CO_2_.

### 2.2. Chemical Reagents

Dimethyl sulfoxide (DMSO) and platelet-derived growth factor (PDGF) were purchased from Beyotime Biotechnology; 2-amino-ethoxydiphenyl borate (2-APB) and nifedipine were from Sigma-Aldrich; thapsigargin and U73122 were from MedChemExpress (Shanghai, China); Ruthenium red (RuR) was from Macklin (Shanghai, China); fibronectin and the calcium-free culture medium were from Thermo Fisher Scientific.

### 2.3. Construction of Calcium FRET Plasmids

The four calcium FRET biosensor plasmids were the cytoplasmic calcium (Cyto-Ca^2+^), ER calcium (ER-Ca^2+^), mitochondria outer membrane calcium (Out-Mito-Ca^2+^), and mito-matrix calcium (Mito-Ca^2+^) biosensors (listed in Figure 1A). For the Cyto-Ca^2+^ biosensor, the FRET expression plasmid used the pcDNA3.1-Ca^2+^-YPet version reported in our previous work [34].

For the ER-Ca^2+^ biosensor, the expression plasmid was generated using the pcDNA3.1-Ca^2+^-YPet and pRSETb-Ca^2+^-YPet plasmids as templates. Through the PCR primer design, the DNA sequence of the ER-targeting signal peptide (MLLPVLLLGLLGAAAD) [35] was added to the 5′ end of the forward primer behind the *Hind III* restriction enzyme site, while the ER retention peptide (KDEL) sequence was added to the 5′ end of the reverse primer before the *EcoR I* site. The DNA fragment, which contained the Ca^2+^-YPet portion along with the two signal peptides, was amplified by PCR using the pRSETb-Ca^2+^-YPet plasmid as the template. After the PCR product was purified using a gel extraction kit (Vazyme, DC301-01), the fragment “ER-ECFP-CaM-M13-YPet-KDEL” and the pcDNA3.1-Ca^2+^-YPet plasmid were both double-digested using *Hind III* and *EcoR I.* The digested PCR fragment and pcDNA3.1 vector were ligated together to generate the ER-Ca^2+^ biosensor.

Similarly, for the Out-Mito-Ca^2+^ biosensor, the signal peptide DAKAP1-encoding sequence [36] was added to the 5′ end of the forward primer behind *Hind III*. The DAKAP1-ECFP-CaM-M13-YPet fragment was amplified by PCR and ligated into the pcDNA3.1 vector after digestion with *Hind III* and *EcoR I* to generate the Out-Mito-Ca^2+^ biosensor.

For the Mito-Ca^2+^ biosensor, we obtained the mitochondrial matrix-targeting signal DNA fragment via a gene synthesis service. It contained the signal peptide sequence as four folds of COX8 (4xMSVLTPLLLRGLTGSARRLPVPRAKIHSLGDP) and a linker peptide (RSGSAKDPT) [37]. The 4xCOX8-linker fragment and pcDNA3.1-ECFP-CaM-M13-YPet were double-digested with *Nhe I* and *Hind III* and ligated together to generate the Mito-Ca^2+^ biosensor.

### 2.4. FRET Plasmid Transfection

According to the instructions for Lipofectamine 3000 (Invitrogen, Waltham, MA, USA), the appropriate number of ASM cells was incubated in a 12-well plate overnight in advance, and then the FRET plasmids (1.5 μg per well) were transfected into cells using Lipofectamine 3000 regents. After 8–10 h, the culture medium was replaced with a new low-sugar DMEM medium containing 10% FBS. After 36 h of transfection, the ASM cells were digested with the Accutase Cell Dissociation Reagent (Thermo) and inoculated into a confocal dish (NEST, Palo Alto, CA, USA) precoated with fibronectin. Then, the cells were subjected to starvation treatment in a medium containing 1% FBS for 16–20 h. Subsequently, FRET imaging experiments were conducted.

### 2.5. Intracellular FRET Imaging of Calcium Biosensors

The Zeiss microscope imaging system has a multi-position function and is equipped with a cell culture chamber (Zeiss, Jena, Germany). In the FRET microscope system, the filter parameters of the ECFP and FRET channels are excitation (436 ± 10 nm), dichroic mirror (455 nm), and emission (480 ± 20 nm) for ECFP, and emission (535 ± 15 nm) for YPet. In our imaging experiments, an ×100 Oil objective was chosen to acquire the FRET images, and the ASM cell samples were placed in the cell culture chamber. By controlling the fast switching between the ECFP and FRET channels through the Zeiss software system (ZEN 2.3 SP1, blue edition), the image data of the ASM cells from both channels were collected almost simultaneously. During the experimental process, fluorescence images were collected at intervals of 1 min for a 20 min duration.

Inhibitor pretreatments included adding appropriate concentrations of reagents such as DMSO (<0.1% *v*/*v*), 2-APB (100 μM), nifedipine (10 μM), and thapsigargin (10 μM) or RuR (10 μM) in advance, followed by incubation for 1 h before cell imaging. To add PDGF stimulation (50 ng/mL) in the middle, 1 mL medium containing PDGF was injected into the dish through a guiding microtube without disruption of the imaging process. In the experimental group with a calcium-free culture, L-glutamine (0.4 mL) and sodium pyruvate (0.2 mL) were added to a calcium-free medium (19.4 mL) in advance. Before performing live cell imaging, the culture medium was removed and washed three times with PBS (phosphate-buffered saline), and then the pre-prepared calcium-free culture medium was added immediately before being placed on the microscope.

### 2.6. Data Processing

The quantitative analysis of image data was conducted using the FRET image analysis software FluoCell 6.0.0 [38]. Principally, after background subtraction, the ratio of the fluorescence intensity was calibrated pixel to pixel between the ECFP and FRET channels, and ratiometric images were acquired along with the FRET/ECFP ratio data. The statistical analysis was conducted in the GraphPad Prism 6.0 software. The time-course curves of the FRET ratio (mean ± S.E.M.) and the graphs with scattering dots (mean ± S.D.), along with the differences between each of the data groups, were analyzed.

Student’s *t*-tests were conducted between the control group and an experimental group, and multiple rounds of *t*-tests were conducted for the various experimental conditions. *, **, ***, and **** indicate *p* values < 0.05, 0.01, 0.001, and 0.0001 to denote significant differences, while ‘ns’ indicates no significant difference.

## 3. Results

### 3.1. Extracellular Calcium Entry Affects Both Calcium Upregulation in Cytoplasm and Calcium Release from ER Storage

When asthma attacks, it is accompanied by the narrowing of the airway and shortness of breath. PDGF is an important growth factor that induces ASM cells’ proliferation and migration into the epidermal layer [9]. Studies have shown that PDGF can cause a significant increase in the calcium concentration in the cytoplasm [2]. Here, we used PDGF as an inducer and a FRET Cyto-Ca^2+^ biosensor for the real-time monitoring of the calcium level in the cytosol of ASM cells. As shown in Figure 1B,D (the time course shown in Movie S1), after stimulation with PDGF, the calcium ions in the cell cytosol [Ca^2+^]_C_ were detected with a rapid increase by the Cyto-Ca^2+^ biosensor. PLC plays an important role in promoting calcium release from the ER via the IP_3_-IP_3_R pathway, and U73122 is a common PLC inhibitor. By pretreating the ASM cells with U73122, the PDGF-induced upregulation of cytoplasmic calcium signaling in the cells was inhibited to a certain extent (Figure 1B,D). This indicates that the PLC pathway plays a role in the calcium signaling induced by PDGF.

To check whether the cytoplasmic calcium increase depends on the calcium flow through the plasma membrane channels from the medium, we studied the effect with a calcium-free culture medium. The [Ca^2+^]_C_ increase induced by PDGF was severely inhibited when examined without calcium in the medium (Figure 1C,D). After pretreating the ASM cells with U73122, the PDGF-induced [Ca^2+^]_C_ was further reduced in the calcium-free medium (Figure 1C,D). Statistical quantifications confirmed that the PDGF-induced cytoplasmic calcium levels and the percentage changes were regulated by both the PLC signal and calcium influx from the medium (Figure 1E,F).

The endoplasmic reticulum (ER) is the largest calcium storage site in cells and contributes the most to the rapid changes in the cytoplasmic calcium levels [10]. By targeting the calcium biosensor in the ER, it is possible to monitor the calcium levels in the subcellular organelles. Here, we attempted to investigate the calcium exchange among the cell cytosol, the ER, and the extracellular medium by using the ER-Ca^2+^ biosensor. As shown in Figure 1G,I (the time course in Movie S2), PDGF stimulation can lead to a dramatic decrease in the ER calcium concentration [Ca^2+^]_ER_, corresponding to the [Ca^2+^]_C_ increase (Figure 1B,C). In comparison, the pretreatment of cells with U73122 resulted in a greater decrease in [Ca^2+^]_ER_ (Figure 1G,I), indicating that the pre-inhibition of the PLC pathway reduced the concentration of free calcium in the ER. In the calcium-free culture medium, the decreasing rate of [Ca^2+^]_ER_ was apparently reduced in comparison to the normal culture medium, corresponding to the reduced increase in cytoplasmic calcium (Figure 1H,I). Statistical quantifications demonstrated that, under the calcium-free medium, the calcium level in ER storage was higher and the change rate was lower in comparison to that under the normal culture (Figure 1J,K). Surprisingly, the inhibition of PLC signaling reduced both [Ca^2+^]_C_ and [Ca^2+^]_ER_ (Figure 1D,E,I,G), which may have resulted from the inhibited extracellular calcium flow through the plasma membrane. These data demonstrate that the PDGF-induced [Ca^2+^]_C_ increase requires both extracellular calcium influx and calcium release from ER storage, while ER calcium release is strongly dependent on extracellular calcium influx.

### 3.2. PDGF-Induced Increase in Cytoplasmic Calcium Is Regulated by ER Calcium Release and Extracellular Calcium Flow through Their Calcium Channels

Regarding the crucial role of calcium exchange between different compartments in mediating the cytoplasmic calcium level, we checked the involvement of several common calcium channels, such as the L-type calcium channels on the plasma membrane (nifedipine as inhibitor), the IP_3_R calcium channel (2-APB), and the SERCA calcium pump (thapsigargin) on the ER membrane.

**Figure 2:**
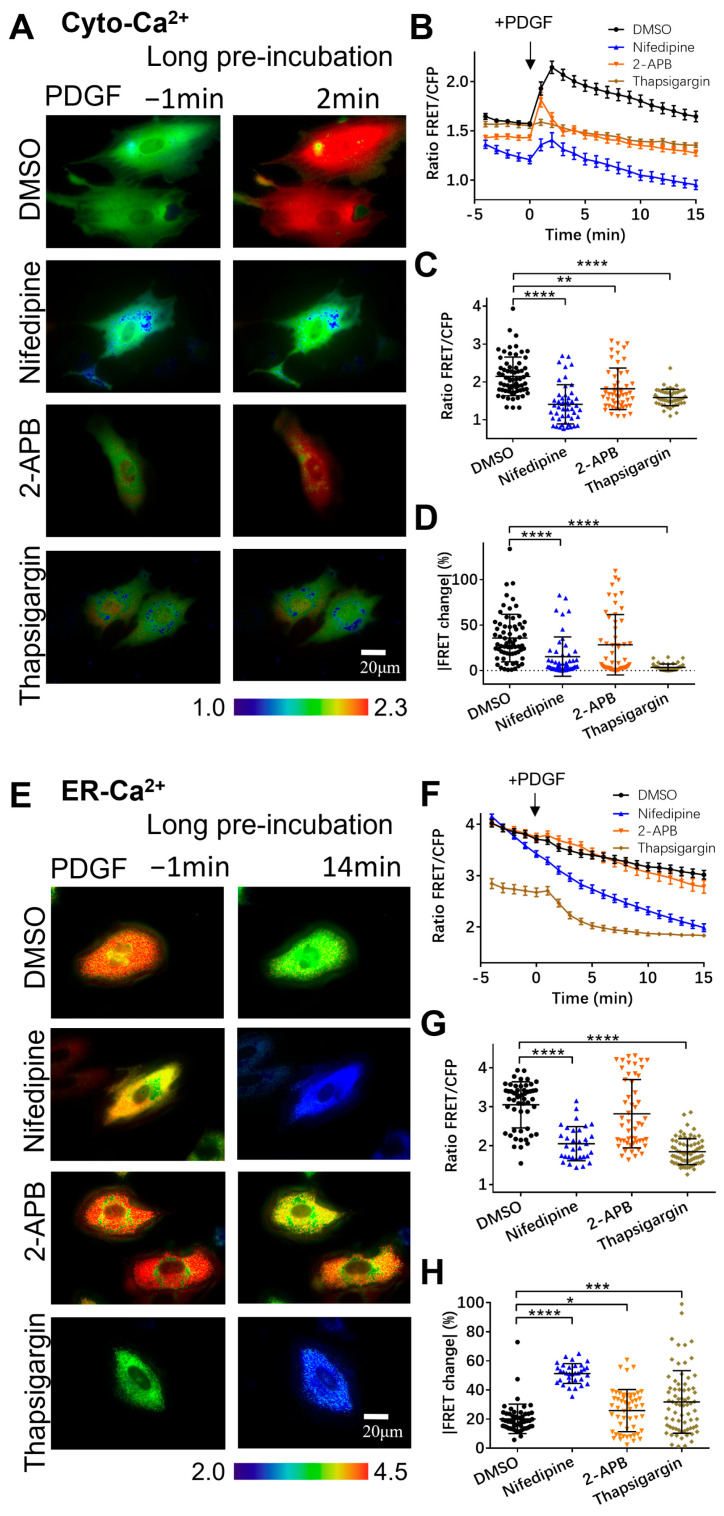
PDGF-induced calcium changes in cell cytoplasm and ER storage with one-hour pre-incubation of calcium channel inhibitors. (**A**) Ratiometric FRET images of Cyto-Ca^2+^ biosensor induced with PDGF in ASM cells pretreated for one hour with DMSO (as control), 2-APB (100 μM), nifedipine (10 μM), and thapsigargin (10 μM). (**B**) Quantified time-course curves of cytoplasmic calcium FRET ratio (FRET/ECFP) in ASM cells under (**A**) conditions. (**C**,**D**) Statistical comparisons of peak values of FRET/ECFP ratio (**C**) and FRET change rates (**D**) from quantified curves in (**B**). Sample sizes of Cyto-Ca^2+^ FRET measurements for DMSO, 2-APB, nifedipine, and thapsigargin are 66, 53, 49, 51, respectively. (**E**) Ratiometric FRET images of ER-Ca^2+^ biosensor induced with PDGF in ASM cells pretreated with DMSO, 2-APB, nifedipine, and thapsigargin. (**F**–**H**) Quantified time-course curves of ER calcium FRET ratio (**F**) and statistical comparisons of peak values of FRET/ECFP ratio (**G**) and FRET change rates (**H**) under various conditions of (**E**). Sample sizes of ER-Ca^2+^ FRET measurements for DMSO, 2-APB, nifedipine, and thapsigargin are 53, 52, 50, 71, respectively. *, **, ***, and **** indicate *p* values < 0.05, 0.01, 0.001, and 0.0001 to denote significant differences.

In comparison with the control group, the cells pretreated with nifedipine for one hour showed the strong inhibition of the increase in [Ca^2+^]_C_ induced by PDGF stimulation (Figure 2A–C), similar to the observations in the calcium-free medium (Figure 1C,D). This result confirms that the calcium flow through the plasma membrane has an essential role in the cytoplasmic calcium increase. The prior inhibition of the ER IP_3_R channel with 2-APB or the ER SERCA pump with thapsigargin also substantially inhibited the [Ca^2+^]_C_ increase (Figure 2A–C). Particularly with SERCA inhibition, the cytosolic calcium response to PDGF stimulation almost disappeared (Figure 2A–C). These data indicate that calcium release from ER storage is critical for PDGF-induced cytoplasmic calcium.

Correspondingly, we also checked the effects of these channel inhibitors on PDGF-induced ER calcium release. Compared to the control group, the ASM cells pretreated with nifedipine showed extremely low [Ca^2+^]_ER_ after PDGF stimulation (Figure 2E,F), indicating that the ER calcium was nearly emptied to supply cytoplasmic calcium when the extracellular calcium flow was inhibited. [Ca^2+^]_ER_ was similar to the control group when the IP_3_R channel on the ER was inhibited by 2-APB (Figure 2E,F). However, [Ca^2+^]_ER_ became extremely low when inhibiting the SERCA pump on the ER (Figure 2E,F), indicating that the active uptake of calcium was essential to maintain the ER’s calcium storage and supply cytoplasmic calcium. These observations were further confirmed by statistical comparisons of the ER calcium FRET levels at 14 min and the percentages of FRET changes after PDGF stimulation (Figure 2G,H). Therefore, the active calcium exchange among the extracellular medium, cell cytosol, and ER storage is crucial for appropriate calcium signal mediation in cells.

**Figure 3:**
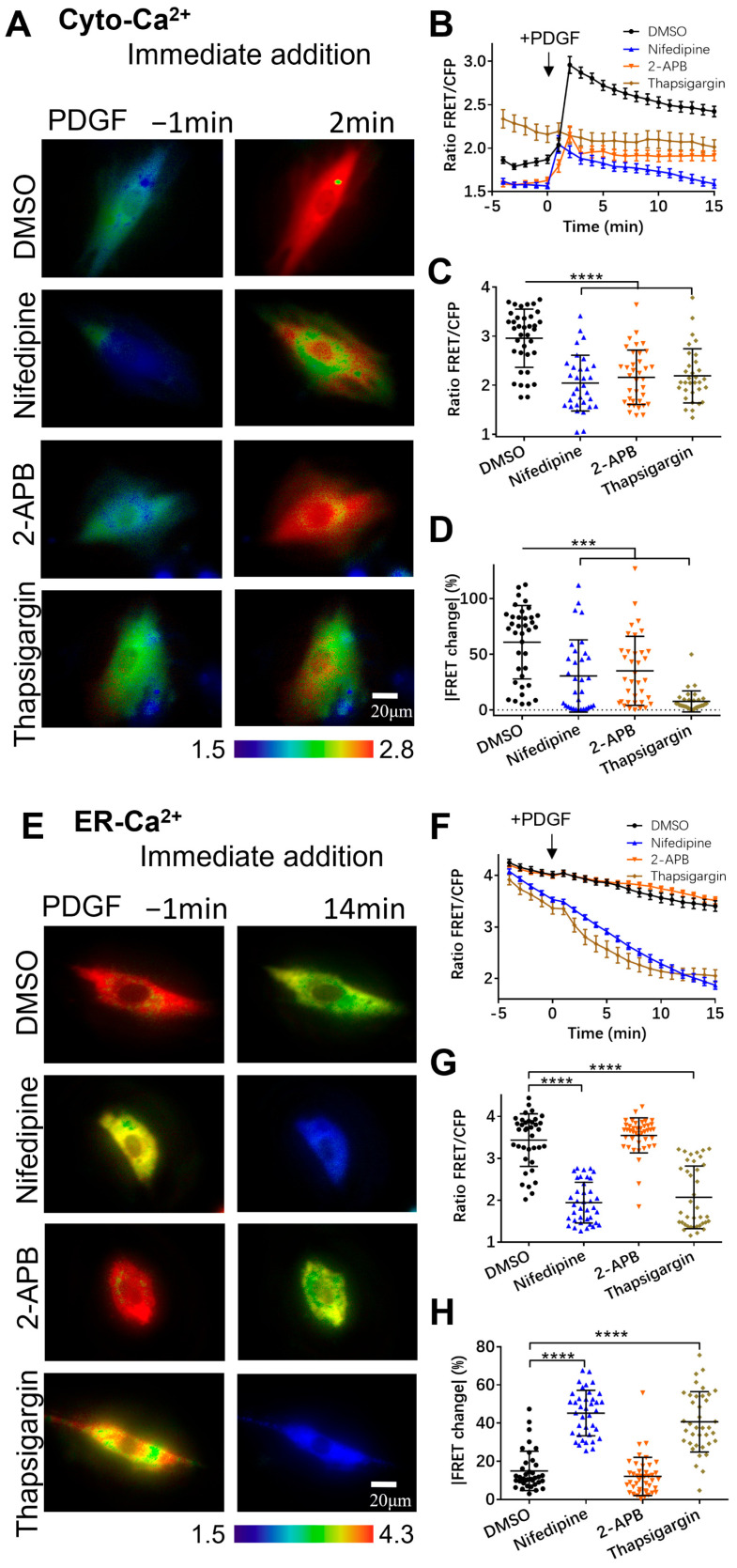
PDGF-induced calcium changes in cell cytosol and ER storage under immediate addition of calcium channel inhibitors before microscopic imaging. (**A**–**D**) Ratiometric FRET images of Cyto-Ca^2+^ biosensor induced with PDGF in ASM cells with immediate addition of DMSO (as control), 2-APB (100 μM), nifedipine (10 μM), and thapsigargin (10 μM) (**A**); corresponding quantified time-course curves of cytoplasmic calcium FRET ratio (FRET/ECFP) (**B**); and statistical comparisons of peak values of FRET ratio (**C**) and FRET change rates (**D**). Sample sizes of Cyto-Ca^2+^ FRET for DMSO, 2-APB, nifedipine, and thapsigargin are 37, 36, 32, 32, respectively. (**E**–**H**) Ratiometric FRET images of ER-Ca^2+^ biosensor induced with PDGF in ASM cells with immediate addition of DMSO, 2-APB, nifedipine, and thapsigargin (**E**); quantified time-course curves of ER calcium FRET ratio (**F**); and statistical comparisons of peak values of FRET/ECFP ratio (**G**) and FRET change rates (**H**). Sample sizes of ER-Ca^2+^ FRET for DMSO, 2-APB, nifedipine, and thapsigargin are 37, 42, 38, 38, respectively. ***, and **** indicate *p* values < 0.001, and 0.0001 to denote significant differences.

Given that one-hour pre-incubation with the inhibitors remarkedly changed the basal [Ca^2+^]_ER_ in the ER storage (Figure 2E,F), we examined the effects of these inhibitors upon immediate addition before FRET imaging. In comparison to the long pre-incubation period, PDGF-induced [Ca^2+^]_C_ showed similar changes under immediate addition (Figure 3A–D). Interestingly, the immediate inhibition of the SERCA pump with thapsigargin caused a basal increase in [Ca^2+^]_C_, implying the inhibitory flux of cytosolic calcium back into the ER storage (Figure 3A,B and Figure S1). A long period of incubation with thapsigargin would reasonably lead to calcium release into the extracellular medium, as a constant high level of [Ca^2+^]_C_ is not normal for cells. The PDGF-induced ER calcium changes also showed similar trends with the immediate addition of the inhibitors in comparison to one-hour pre-incubation (Figure 3E–H). The basal [Ca^2+^]_ER_ in the ER storage with the immediate addition of thapsigargin was less different from the control group (Figure 3E,F) in comparison to the remarkedly changed basal [Ca^2+^]_ER_ under one-hour pre-incubation conditions (Figure 3A,B).

### 3.3. Regulation of Mitochondrial Calcium by Calcium Channels

Mitochondrial organelles are also important in regulating calcium signals and thus maintaining calcium homeostasis for physiological processes, including ATP production [39]. Calcium uptake occurs mostly through the mitochondrial calcium uniporter complex (MCU), consisting of multiple subunits [40]. We further generated mitochondria-targeting biosensors to investigate the calcium exchange between mitochondria and the calcium pools. Adding signal peptides to the Cyto-Ca^2+^ biosensor, we developed two versions by localizing the biosensor in the mitochondrial matrix (Mito-Ca^2+^) and on the outer mitochondrial membrane (Out-Mito-Ca^2+^) [36,37] (Figure 1A).

**Figure 4:**
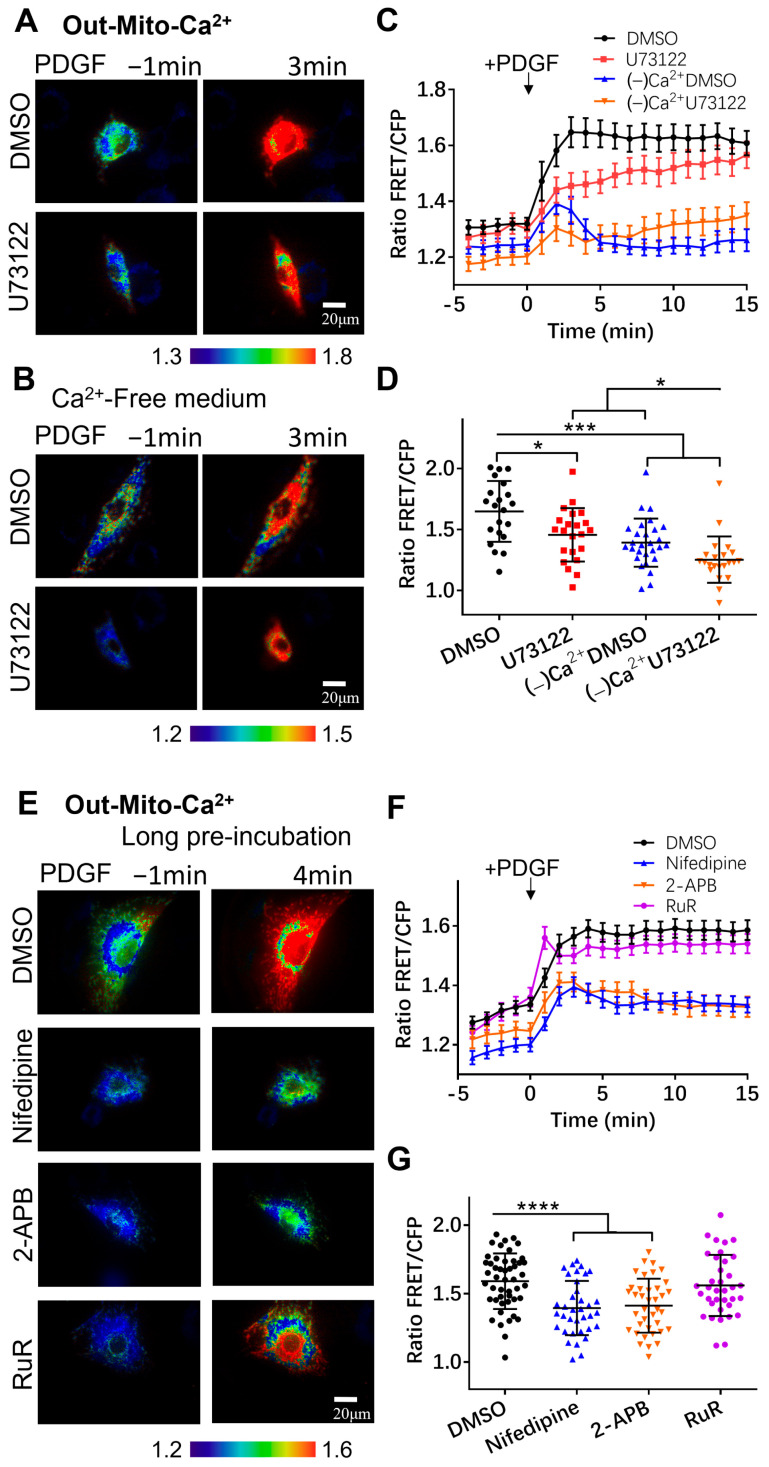
PDGF-induced calcium changes at the outer mitochondrial membrane measured by the Out-Mito-Ca^2+^ FRET biosensor. (**A**,**B**) PDGF-induced ratiometric FRET images of ASM cells pretreated with DMSO or U73122 (10 μM) in normal culture medium (**A**) or calcium-free medium (**B**). (**C**,**D**) Quantified time-course curves of Out-Mito-Ca^2+^ FRET ratio (**C**), and statistical comparisons of peak values of FRET/ECFP ratio (**D**) under (**A**,**B**) conditions. Sample sizes for DMSO, U73122, (−)Ca^2+^/DMSO, and (−)Ca^2+^/U73122 are 33, 33, 40, 33, respectively. (**E**) Ratiometric FRET images of Out-Mito-Ca^2+^ biosensor induced by PDGF in ASM cells with one-hour pre-incubation of DMSO, 2-APB (100 μM), nifedipine (10 μM), and RuR (10 μM). (**F**,**G**) Quantified time-course curves of Out-Mito-Ca^2+^ FRET ratio (**F**) and statistical comparisons of peak values of FRET/ECFP ratio (**G**). Sample sizes of Out-Mito-Ca^2+^ FRET for DMSO, 2-APB, nifedipine, thapsigargin, and RuR are 48, 38, 36, 36, respectively. *, ***, and **** indicate *p* values < 0.05, 0.001, and 0.0001 to denote significant differences.

The outer mitochondrial membrane faces the cytoplasm, and the Out-Mito-Ca^2+^ biosensor measured largely similar changes in calcium levels to the Cyto-Ca^2+^ one. As shown in Figure 4A,C, PDGF induced a calcium increase at the outer mitochondrial membrane sub-region ([Ca^2+^]_OM_), displaying a sustained Ca^2+^ signal, and the inhibition of the PLC pathway with U73122 had little effect. When the ASM cells were in the calcium-free medium, the [Ca^2+^]_OM_ increase was markedly reduced, and it was almost completely inhibited by the further addition of U73122 (Figure 4B,C), similar to the responses of cytosolic [Ca^2+^]_C_ (Figure 1B–D). These observations were confirmed by statistical quantifications and comparisons among these experimental conditions (Figure 4D). When we inhibited the membrane L-type or ER IP_3_R calcium channels with nifedipine or 2-APB, the PDGF-induced [Ca^2+^]_OM_ increase measured by the Out-Mito-Ca^2+^ biosensor was markedly reduced, in contrast to the control group (Figure 4E–G, Movie S3). The inhibition of the MCU channel on mitochondria with Ruthenium red (RuR) did not change the PDGF-induced [Ca^2+^]_OM_ increase (Figure 4E–G). Hence, the PDGF-induced calcium level at the outer mitochondrial membrane sub-region showed a similar regulatory response to the cytosolic one.

We further attempted to measure the calcium level within the mitochondrial matrix (mito-matrix) using the Mito-Ca^2+^ biosensor. In our experiments, the mito-matrix-localized biosensor protein did not achieve bright fluorescence as in the other three versions. We do not present the time courses of the FRET data as the images did not achieve the same quality as the others obtained in this work.

### 3.4. Characterization of Calcium Exchange in Cellular Compartments

**Figure 5:**
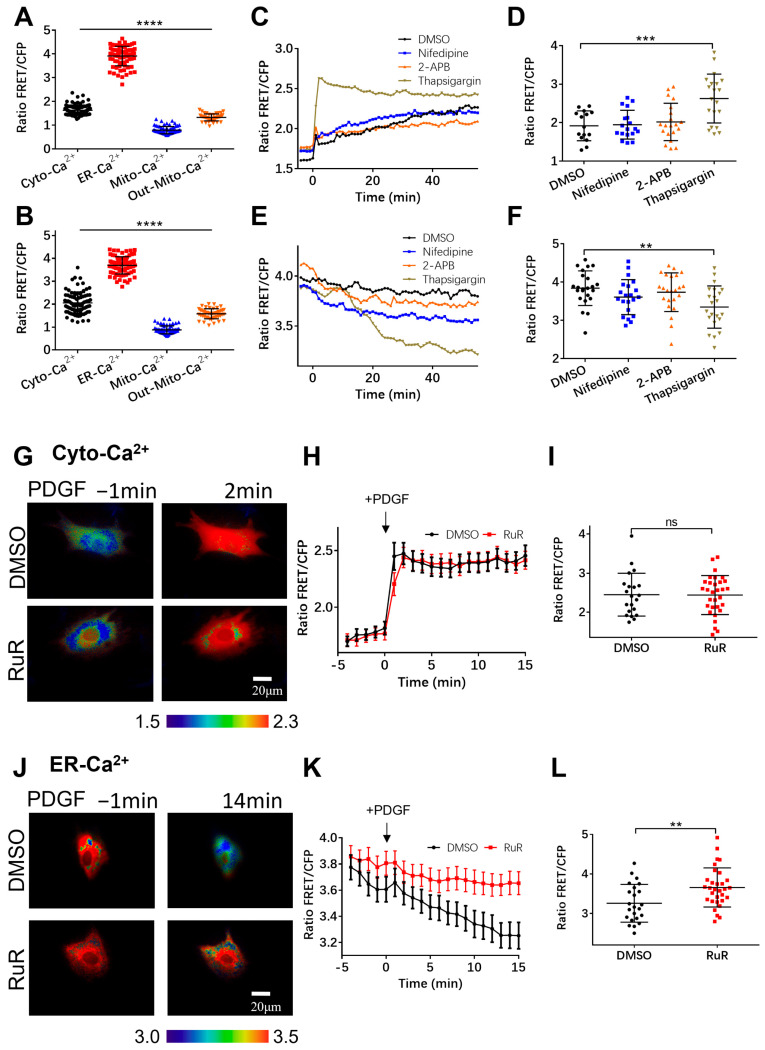
Characterization of calcium levels in cellular compartments. (**A**,**B**) Statistical comparisons of calcium FRET levels in cellular cytoplasm (Cyto-Ca^2+^), ER (ER-Ca^2+^), and outer mitochondrial membrane (Out-Mito Ca^2+^ in resting ASM cells (**A**) or with PDGF stimulation (at 5 min)) (**B**). The ratio values and sample sizes for cases without/with PDGF stimulation: Cyto-Ca^2+^ (1.6 ± 0.022, N = 87)/(2.05 ± 0.05, N = 87), ER-Ca^2+^ (3.9 ± 0.046, N = 78)/(3.7 ± 0.043, N = 78), and Out-Mito-Ca^2+^ (1.3 ± 0.021, N = 48)/(1.58 ± 0.032, N = 48), respectively. (**C**–**F**) The time courses (average values) and FRET ratio comparisons of cytosolic calcium (peak values) (**C**,**D**) and ER (at 25 min) (**E**,**F**) calcium levels in ASM cells treated with DMSO, nifedipine, 2-APB, and thapsigargin. (**G**–**I**) Ratiometric FRET images of Cyto-Ca^2+^ biosensor induced with PDGF (**G**), time courses of cytosolic calcium FRET ratio (**H**), and statistical comparison of peak values of FRET/ECFP ratio (**I**) in ASM cells pretreated with DMSO or RuR (10 μM). (**J**–**L**) Ratiometric FRET images of ER-Ca^2+^ biosensor induced with PDGF (**J**) and time courses (**K**) and statistical comparison (at 14 min) of FRET/ECFP ratio (**L**) in ASM cells pretreated with DMSO or RuR (10 μM). **, ***, and **** indicate *p* values < 0.01, 0.001, and 0.0001 to denote significant differences.

The calcium concentrations are inhomogeneous in cells [41,42]. The subcellular-located calcium biosensors allow us to measure the calcium levels and study the regulatory mechanisms in the different cellular compartments. Our measurements using the FRET biosensors showed that the ER had the highest calcium level, where the FRET ratio reached 3.9 ± 0.046, followed by the cytosolic calcium (1.6 ± 0.022) and mitochondrial outer membrane calcium (1.3 ± 0.021), while the mitochondrial matrix calcium was the lowest (0.79 ± 0.018) among them (Figure 5A). This contrasting trend among the cellular compartments was maintained after PDGF stimulation for 5 min (Figure 5B). It should be noted that the FRET ratio and calcium concentration do not show a linear relationship. The data indicated that mitochondrial calcium had its own regulatory mechanism, and it showed a lower calcium level compared to the cytosolic one.

We further applied inhibitors of calcium channels to check the calcium flow between the subcellular compartments without PDGF stimulation. It was found that the inhibition of ER calcium uptake with thapsigargin (ER calcium pump inhibitor) caused a rapid increase in [Ca^2+^]_C_ (Figure 5B,C), which corresponded to a sharp decrease in [Ca^2+^]_ER_ (Figure 5D,E). The inhibition of the L-type calcium channel on the plasma (by nifedipine) and the IP_3_R channel on the ER (by 2-APB) did not cause significant differences in the changes in the calcium levels compared to the control group (Figure 5B–E). Hence, the calcium exchange between the cytosol and ER storage is very dynamic and active in cells.

We also examined the inhibition of the mitochondrial calcium uniporter (MCU) channel in mitochondria with Ruthenium red (RuR) to check the calcium exchange between the mitochondria and the cytoplasm. The inhibition of the MCU channel did not cause significant differences in the changes in the cytosolic calcium level [Ca^2+^]_C_ compared to the control group (Figure 5F–H), whereas RuR treatment reduced the calcium release from the ER storage with PDGF stimulation (Figure 5I–K). It has been reported that RuR also inhibits the ryanodine receptor (RyR) located on the ER for calcium release [43], which is consistent with our observations (Figure 5I–K). Therefore, mitochondria take up calcium through their own calcium channels and maintain a relatively low calcium level. We did not find evidence showing a Ca^2+^ pool within the mitochondria to regulate the Ca^2+^ level in the cytoplasm.

In addition to the FRET images in Figure 1, Figure 2, Figure 3, Figure 4 and Figure 5, we provide three more representative images for each experimental group in Figures S2–S6, respectively. The multiple cell samples demonstrated similar trends in the FRET response for each condition.

## 4. Discussion

Intracellular calcium homeostasis is extremely important and closely related to many cellular physiological processes [44,45,46]. PDGF can also regulate physiological processes such as cell proliferation, migration, and differentiation by altering the intracellular calcium levels [9]. We investigated the PDGF-induced transport of intracellular calcium among different cellular compartments by generating calcium FRET biosensors tagged with subcellular-targeted peptides.

Previous studies have shown that PDGF stimulation increases calcium influx [47]. We confirmed that a PDGF-induced cytosolic calcium increase is also related to the regulation of the ER. Our experiments demonstrated that the extracellular calcium flow through the plasma membrane can affect the calcium regulation of the cytoplasm, ER, and mitochondria. When the calcium in the extracellular environment is depleted, the PDGF-induced [Ca^2+^]_C_ increase in the cytoplasm and the release of calcium from the ER are significantly inhibited (Figure 1). After ER calcium release is reduced, the cytoplasm loses important sources of calcium signaling (Figure 2 and Figure 3). Therefore, the calcium signaling changes caused by PDGF are mainly provided by the extracellular environment and ER, flowing towards the cytoplasm, including the mitochondria.

Cells have a certain self-protection mechanism, and when a calcium overload occurs in the ER, certain pathways are activated to maintain the intracellular calcium homeostasis [48,49]. In addition, we observed changes in the intracellular calcium signal flow after inhibiting IP_3_R, the L-type calcium pathway, and the SERCA calcium pump. The inhibition of IP_3_R can reduce the upregulation of cytoplasmic and mitochondrial calcium caused by PDGF. However, there is no significant inhibitory effect on calcium release from the ER. This may be regulated by another calcium release channel, the ryanodine receptor on the ER membrane. Additionally, our recent work reported that the L-type calcium channel is not relevant to mechanical-stretch-activated ERK via calcium signals [50]. Hence, the chemical and biomechanical activation of intracellular calcium may occur through different sets of calcium channels on the plasma membrane.

After the certain inhibition of calcium influx in cells, the average calcium changes in the cytoplasm and mitochondria decreased significantly, which was consistent with the experimental results in the calcium-free culture medium. Surprisingly, the PDGF-induced ER calcium release was attenuated by the higher reserved [Ca^2+^]_ER_ levels after the immediate switch to the calcium-free culture medium (Figure 1I,J). This indicates that the extracellular calcium influx may enhance the ER release of calcium. This observation can be attributed to the calcium-dependent activation of the ryanodine receptor (RyR), resulting in ER calcium release [51]. Our data prove that extracellular calcium influx effects ER calcium release and is essential for the PDGF-induced full activation of cytosolic calcium signals in cells.

The inhibition of the SERCA calcium pump initially leads to an increase in cytoplasmic calcium levels, but this gradually decreases over time and it becomes unresponsive to PDGF stimulation (Figure 2 and Figure 3) [51]. Our experimental data confirmed the process of calcium release from the ER into the cytosol, where it is further exported into the medium with the addition of a SERCA inhibitor (Figure 5C–F). Hence, the dynamic and active calcium shuttling between the ER storage and cytoplasm is essential to maintain calcium homeostasis in cells.

The calcium level near the outer mitochondrial membrane showed similar responses to PDGF-induced cytosolic calcium, which was not influenced by the mitochondrial MCU channel’s inhibition with RuR (Figure 4). In addition, treatment with an RuR inhibitor did not interfere with the PDGF-induced cytosolic calcium increase, but significantly reduced the ER’s release of calcium (Figure 5G–L), likely through the RuR inhibition of ryanodine receptors on the ER [43]. Hypothetically, extracellular calcium might play a compensatory role regarding the reduced release of cytosolic calcium from the ER with RuR treatment.

## 5. Conclusions

**Figure 6:**
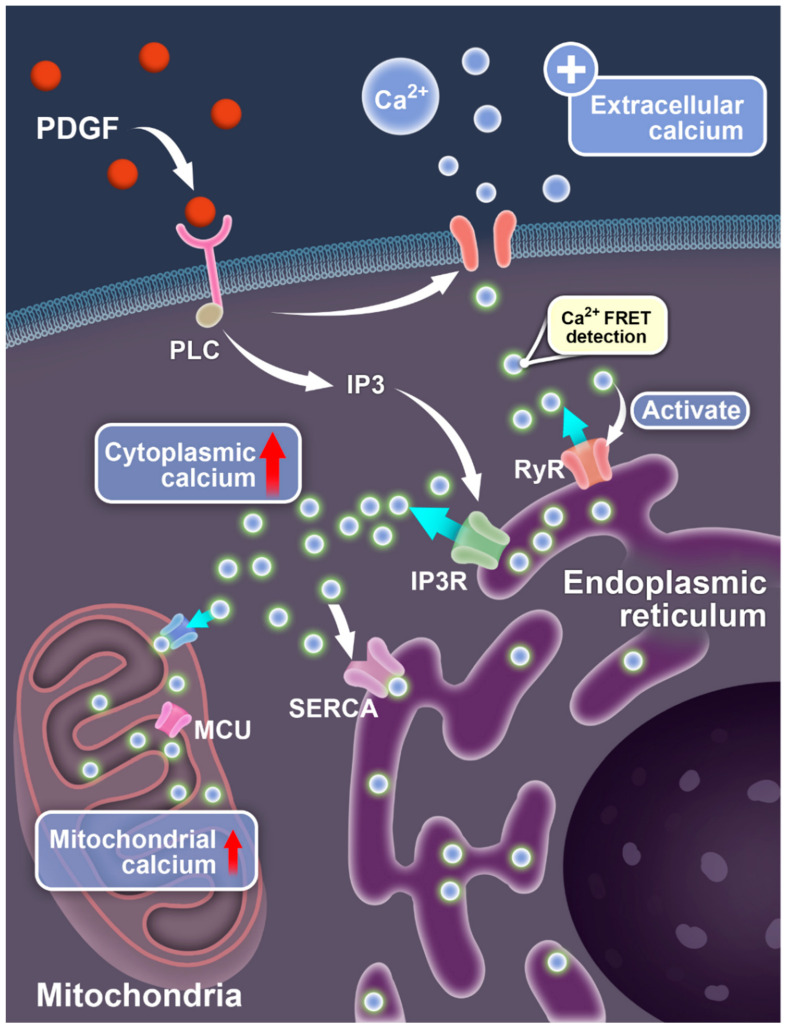
Illustration of PDGF-induced calcium exchange between different compartments of cells. The diagrams show the signaling-mediated Ca^2+^ ion flow pathways between the extracellular medium, cell cytosol, endoplasmic reticulum, and mitochondria.

Based on our study with subcellular-targeted FRET biosensors, along with previous reports, the calcium exchange and regulation among the different compartments of cells can be summarized as shown in Figure 6. As demonstrated in the schematics, PDGF-activated PDGFR signaling mediates the extracellular calcium flow through the plasma membrane (which further activates calcium release from the endoplasmic reticulum (ER) storage as an amplifying effect) and ER calcium release through the PLC-IP_3_-IP_3_R and RyR pathways, which results in a cytosolic [Ca^2+^]_C_ increase. The ER takes up calcium through the SERCA pump to maintain the calcium homeostasis in the cells, and the mitochondria take up calcium through the MCU channel. When there is no calcium in the medium, upon PDGF stimulation, the lack of extracellular calcium entry leads to inhibited ER calcium release and reduced cytosolic [Ca^2+^]_C_ increases.

## Data Availability

Data are contained within the article and supplementary materials.

## Supplementary Materials

The following supporting information can be downloaded at: https://www.mdpi.com/article/10.3390/bios14040179/s1, Figure S1: The basal level comparisons of calcium FRET in cell cytosol or endoplasmic reticulum (ER) after the inhibitors’ treatments but before PDGF stimulations; Figures S2–S6: Multiple representative cell samples for the corresponding FRET images in Figure 1, Figure 2, Figure 3, Figure 4 and Figure 5; Movies S1–S3: Calcium FRET changes at the cytoplasm, ER, and outer mitochondrial membrane of ASM cells induced by PDGF stimulation.
