# Supplementary material for: Characterization of PDGF-Induced Subcellular Calcium Regulation through Calcium Channels in Airway Smooth Muscle Cells by FRET Biosensors
Source: Biosensors (Basel). 2024 Apr 7;14(4):179. doi: 10.3390/bios14040179 (PMC11048225; doi:10.3390/bios14040179)
Supplement: Supplementary file 1 [file biosensors-14-00179-s001.zip › Supplementary materials 2024.03/Supporting information-2024.03.docx]

**Characterization of PDGF-induced subcellular calcium regulations through calcium channels in airway smooth muscle cells by FRET biosensors**

Mingxing Ouyang^1,†,*^, Binqian Zhou^1,2,†^, Chunmei Li^1^, Linhong Deng^1,*^

^1^Institute of Biomedical Engineering and Health Sciences, School of Medical and Health Engineering, Changzhou University, Changzhou, 213164 China

^2^School of Pharmacy, Changzhou University, Changzhou, 213164 China

^†^M.O. and B.Z. are co-first authors.

^*^Corresponding authors.

The supporting information contains one figure and three movies.

**Movie legends:**

**Movie S1.** The time-lapse images of cytosolic calcium FRET in ASM cells before and after PDGF stimulation. The interval is 1 min.

**Movie S2.** The time-lapse images of ER calcium FRET in ASM cells before and after PDGF stimulation. The interval is 1 min.

**Movie S3.** The time-lapse images of calcium FRET on the outer mitochondrial membrane in ASM cells before and after PDGF stimulation. The interval is 1 min.


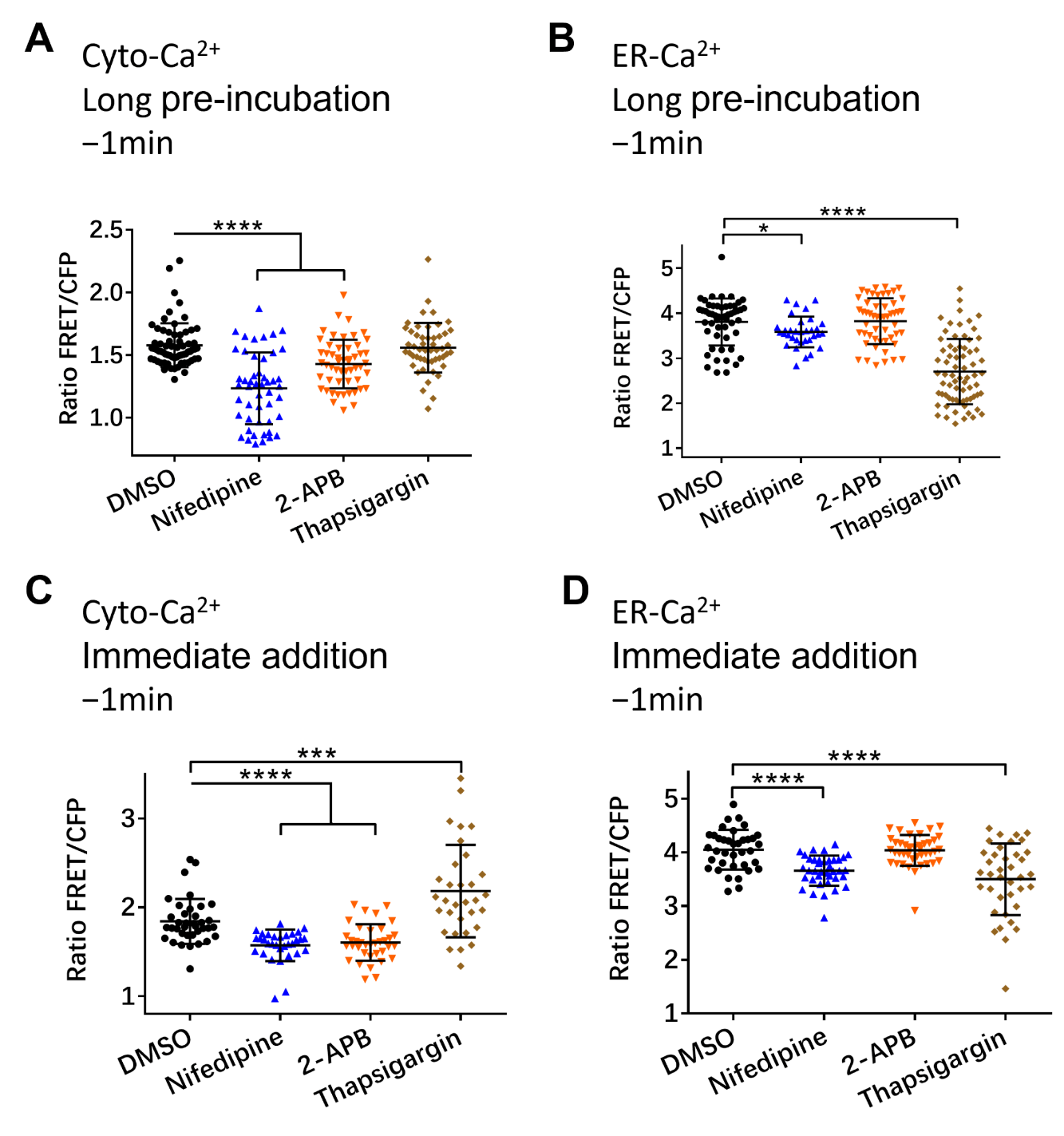


**Figure S1. The basal level comparisons of calcium FRET in cell cytosol or endoplasmic reticulum (ER) after the inhibitors’ treatments but before PDGF stimulations. (A, B)** The calcium FRET levels in the cytosol (A) or ER (B) after one hour pre-incubation with DMSO, nifedipine, 2-APB, or thapsigargin. **(C, D)** The calcium FRET levels in the cytosol (C) or ER (D) from immediate addition of DMSO, nifedipine, 2-APB, or thapsigargin before moved to microscopic imaging.


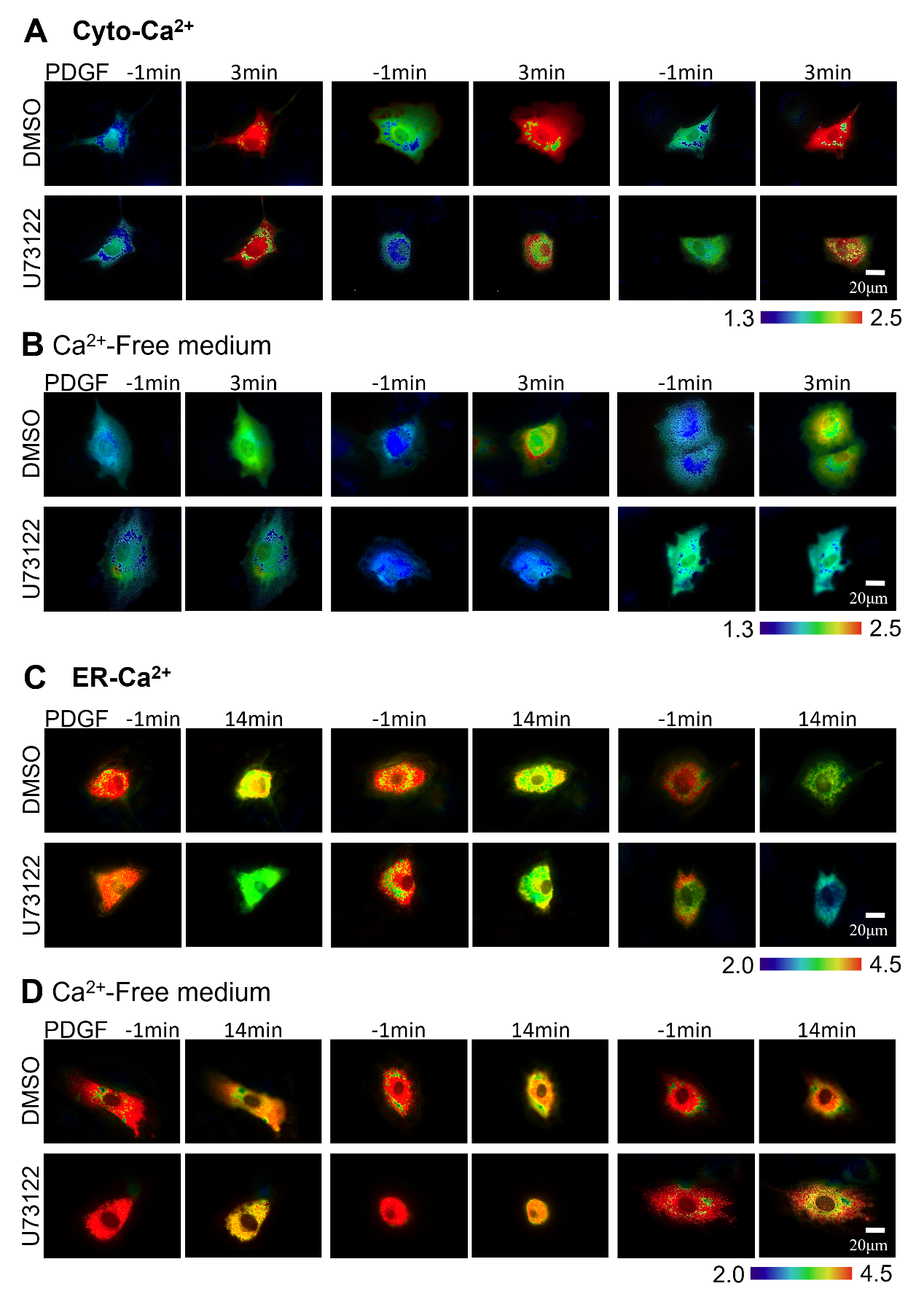


**Figure S2**. Multiple representative cell samples for the corresponding FRET images in Figure 1B, C, G, H.


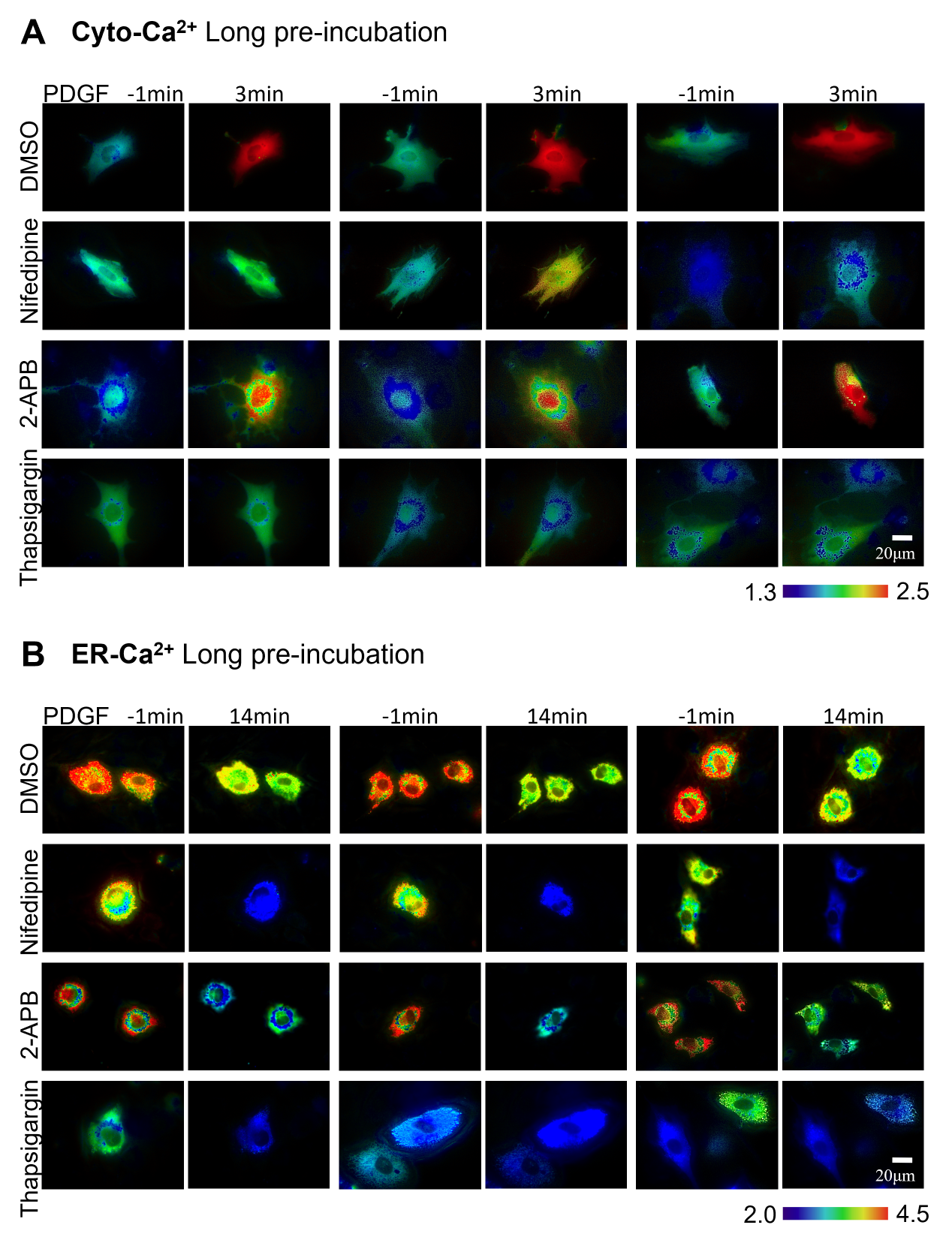


**Figure S3**. Multiple representative cell samples for the corresponding FRET images in Figure 2A, E.


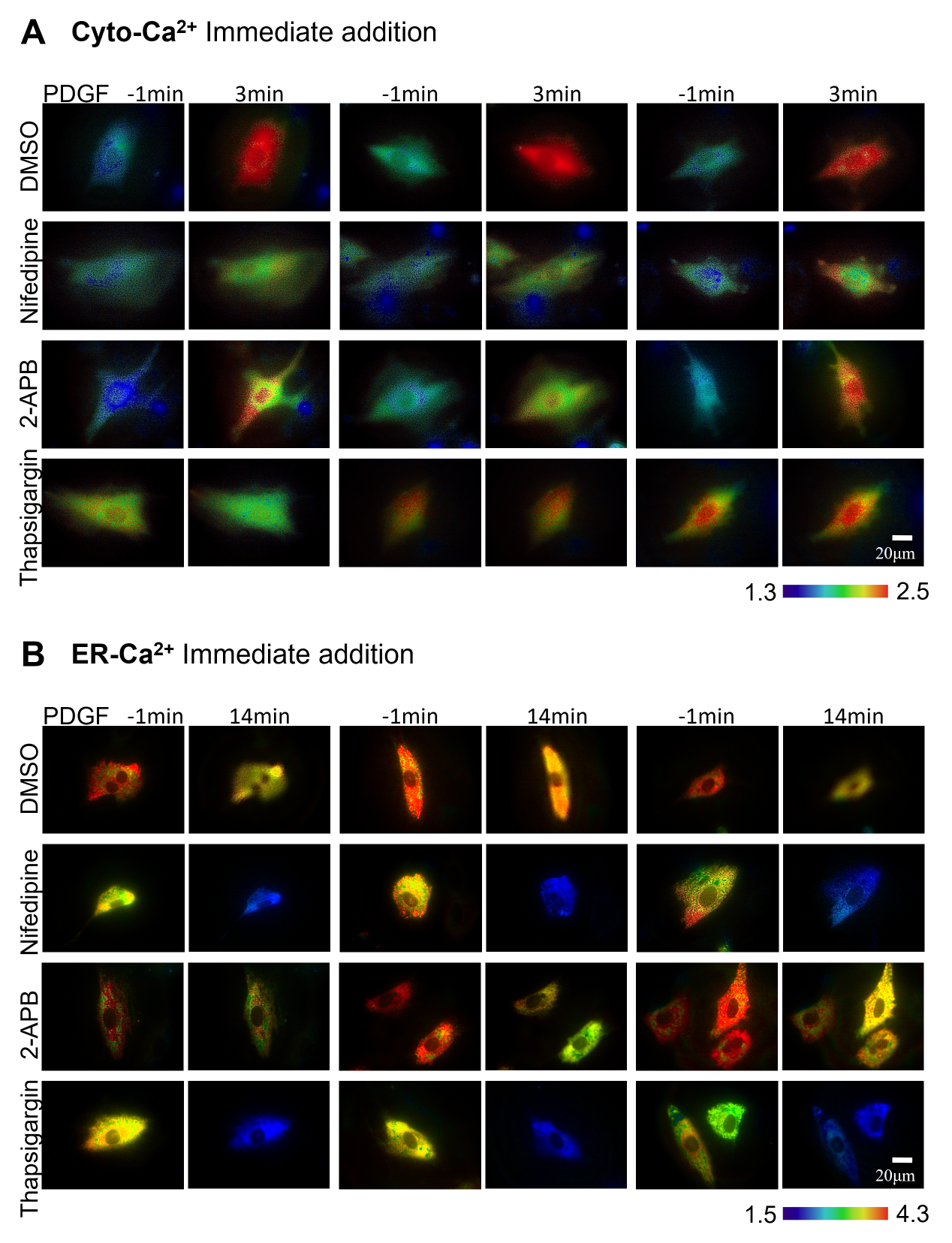


**Figure S4**. Multiple representative cell samples for the corresponding FRET images in Figure 3A, E.


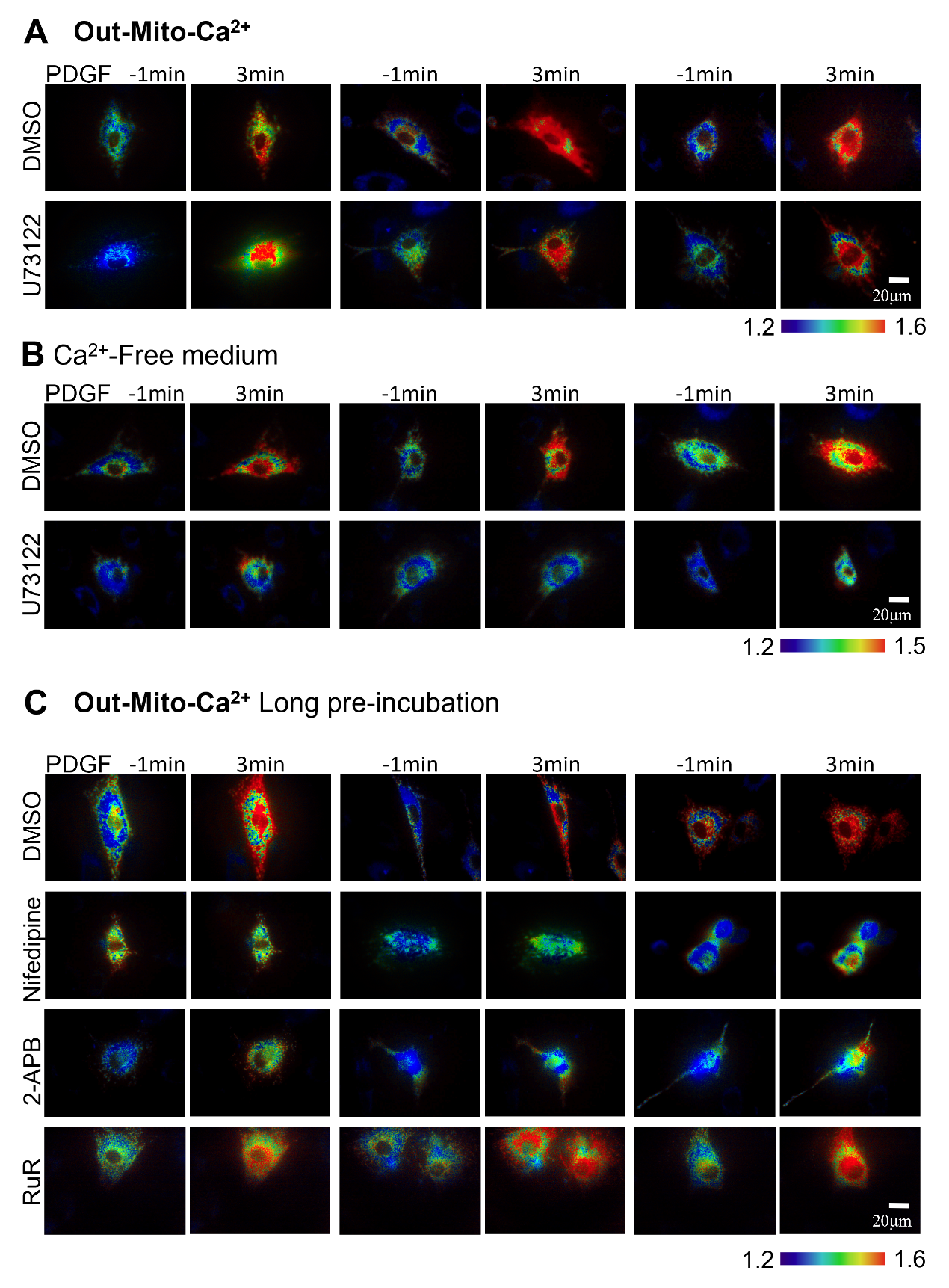


**Figure S5**. Multiple representative cell samples for the corresponding FRET images in Figure 4A, B, E.


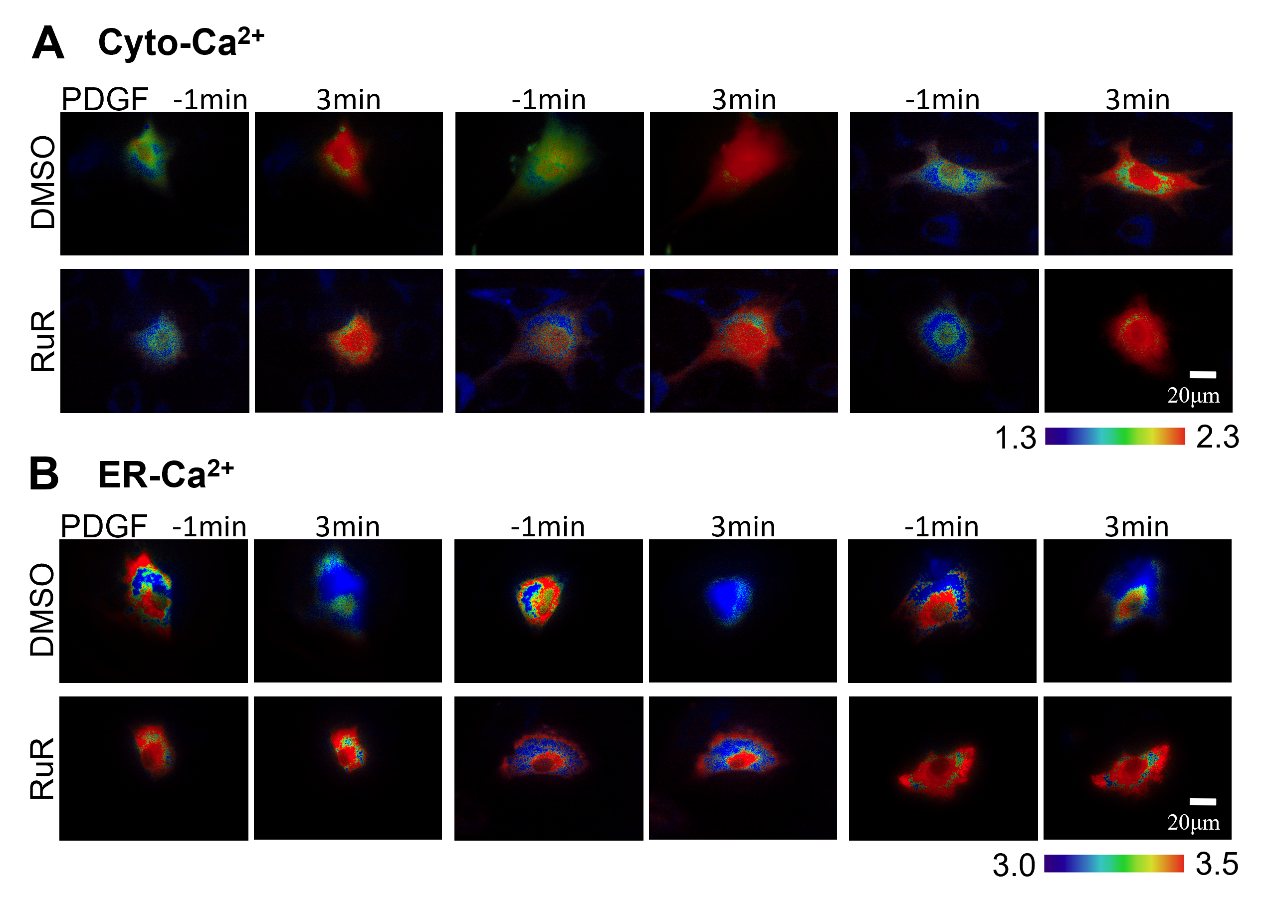


**Figure S6**. Multiple representative cell samples for the corresponding FRET images in Figure 5G, J.
